# The compliance and revenue benefits of ProHeart Vs monthly heartworm disease preventives in the US

**DOI:** 10.1371/journal.pone.0271058

**Published:** 2022-08-11

**Authors:** Kennedy Mwacalimba, Kristine Smith, Marina Moldavchuk, Derek Sears, Christopher Brennan

**Affiliations:** 1 Zoetis Inc, Parsippany, NJ, United States of America; 2 Covetrus, Portland, ME, United States of America; University of Agricultural Sciences and Veterinary Medicine Cluj-Napoca, Life Science Institute, ROMANIA

## Abstract

The aim of the current study was to understand how the canine heartworm disease preventive ProHeart® 12 (extended-release injectable moxidectin, PH 12), impacts heartworm preventive purchase compliance and veterinary practice revenue over time compared to monthly heartworm disease preventives. This was a preliminary observational purchase compliance and revenue study based on a retrospective review of transaction data from 4,615 general practices across the United States. The review period was from September 2018 to August 2020. Anonymous transaction records of over 13 million canine patients were analyzed. Of these, only 3.5 million (25.7%) patients purchased any heartworm preventive, as has been presented in other studies. Practices that implemented PH 12 demonstrated the most growth in canine heartworm prevention revenue, patients, and patient compliance levels during the 12-month observation period, compared to previous year. These practices saw year over year growth in percent patients receiving heartworm protection, as well as 10% and 15% growth in the proportion of preventive patients compliant for more than 6 months and 12 months respectively. In contrast, practices that did not bring on PH 12 and only dispensed monthly heartworm preventives saw a decline in the proportion of canine preventive patients that were compliant for more than 6 months. Similarly, PH 12 practices experienced 15% growth in preventive revenue, and practices that did not bring on PH 12 only experienced 3.9% growth in preventive revenue. PH 12 was single-handedly responsible for all growth in patients compliant for more than 6 months in this study. Growth in protection of canine patients with PH 12 proves a helpful tool where mitigation strategies have thus far failed to curb increasing canine heartworm disease prevalence in the US.

## Introduction

Canine heartworm disease has increased in prevalence and geographical range over the past several decades [1, 2] despite availability of effective monthly heartworm preventives. The American Heartworm Society (AHS) has identified the main cause of preventive failure to be due to poor compliance. AHS guidelines state that dogs should be on a US Food and Drug Administration (FDA) approved heartworm disease preventive year-round [3]. Compliance is a primary focus as even a single missed or delayed preventive dose by owners can lead to canine patient infections. Due to the lifecycle of the canine heartworm, *Dirofilaria immitis*, the L3 larvae from an infected mosquito bite enter the immature adult stage within the canine host by day 50 to 70 and are no longer susceptible to macrocyclic lactone preventives [3, 4].

Compliance with AHS guidelines remain low [1, 5]. Despite decades long availability of macrocyclic lactone preventives, the AHS heartworm surveillance map has shown steady spread of this infectious disease across the country [2], prompting the concern that the veterinary community is losing the battle against this fatal disease. According to one study, veterinarians surveyed reported a 21% increase in canine cases from 2013–2016 alone [1]. Further, resistance to macrocyclic lactones in *D*. *immitis* is being observed, raising concerns of apparent loss of efficacy of this drug class, partially fueled by lack of full prophylactic compliance [6].

ProHeart® 6 (PH 6) and ProHeart® 12 (PH 12) (Zoetis, Parsippany, NJ, USA) were developed to help veterinarians resolve the owner compliance challenge in canine heartworm disease prevention. They employ unique microsphere technology that delivers preventive levels of moxidectin and thus continuous protection against heartworm infection caused by *D*. *immitis*. A single dose of ProHeart 6 (0.17 mg/kg) protects dogs against heartworm disease for 6 months, and ProHeart 12 (0.5 mg/kg) protects dogs for 12 months [7]. Both formulations are also for the treatment of existing larval and adult hookworm (*Ancylostoma caninum* and *Uncinaria stenocephala*) infections. The identical product to PH 12 is already registered in Australia, New Zealand and Japan as ProHeart® SR-12 and has served as the leading heartworm preventive product in Australia for nearly two decades. PH 12 was approved by the FDA in July of 2019.

The aim of the current preliminary observational purchase compliance and revenue study was to understand how PH 12 administration impacted heartworm preventive compliance and practice revenue since its arrival on the US market compared to monthly heartworm preventives, using retrospective transaction data from general practices affiliated with Vetstreet. The review period was from September 2018 to August 2020.

## Methods

This study followed the guidelines and checklist for a systematic approach to compliance and persistence studies using retrospective databases as published by Peterson et al. [8]. Vetstreet veterinary practice management service (Covetrus, Inc, 7 Custom House Street, Portland, ME) has over 6 thousand practices in their US database. Of those, practices were included in this study if they had records of heartworm preventive (HWP) transactions 12 months of the year, with the purpose of excluding practices that were not active in HWP sales. Practices of the over 6 thousand practices that met study inclusion criteria were 4,615. Year over year (YOY) comparisons were made between two annual time periods; Period 1, September 1, 2018 –August 31, 2019 and Period 2, September 1, 2019 –August 31, 2020.

Practices included in this study were deidentified and anonymized by Vetstreet, then divided into exclusive groups according to their HWP pharmacy portfolio. All practices carried and dispensed oral or topical monthly heartworm preventives (MHWP) every month in both periods. Additionally, practices were only considered as users/dispensers of ProHeart 6 (hereafter referred to as PH 6) or PH 12 if they utilized the product (in any amount) for at least 6 months during the year. If practices carried and utilized either PH product for less than 6 months, they were considered non-PH practices. The reason for this was to only include practices that had given time to implement the product into their pharmacy.

Groups were divided as follows:

- Group 1 (“New PH 12 Users”): Practices dispensing monthly heartworm preventive (MHWP) in Period 1 and 2, AND not utilizing PH 6 in Period 1, AND implementing PH 12 in Period 2.
- Group 2 (“PH 6 to PH 12 Users”): Practices dispensing MHWP in Period 1 and 2 AND utilizing PH 6 in Period 1 and Period 2 AND implementing PH 12 in Period 2.
- Group 3 (“PH 6 Only Users”): Practices dispensing MHWP in Period 1 and 2 AND utilizing PH 6 in Period 1 and Period 2, AND not implementing PH 12 in Period 2.
- Group 4 (“Non-PH Users”): Practices dispensing MHWP in Period 1, AND not utilizing PH 6 or PH 12 in either Period 1 or 2.

None of the groups carried PH 12 in Period 1 for more than 6 months, as PH 12 was only released to the market on July 2019 and Period 1 ended in August 2019. Of those practices that did carry PH 12 for less than 6 months in Period 1, it only represented an average1% of their HWP transactions in that Period. However, practices could be carriers of PH 6 in either year since PH 6 has been on the market in the US since 2008.

MHWPs in this study were grouped together, as all monthly heartworm preventives were considered one preventive modality. The study included nine most commonly prescribed heartworm preventive or combination heartworm preventive brands. Following eligibility analysis, Vetstreet prepared an aggregated de-identified summary highlighting the number of practices with transactions for these medications, the volume of these transactions (i.e., monthly dose equivalents sold), the value of these transactions (i.e., gross heartworm preventive revenue from MHWP, PH 6, and PH 12), and the change in the volume and value of these transactions between Period 1 and Period 2. Outputs were descriptive summary statistics (totals, arithmetic means and proportions) generated in Microsoft Excel™ of the following variables: proportion of canine patients receiving heartworm preventive per year, compliance in terms of months of protection, and revenue earned by the practice. The Poisson 95% Confidence Interval for the proportions presented were calculated using MEDCALC® https://www.medcalc.org/calc/rate_ci.php.

## Results

### Patients protected and compliance

#### All groups

All 50 US states were represented by the 4,615 practices meeting study inclusion criteria (practices with records of HWP transactions 12 months of the year) with a total of 25.3% (3,299,200 of 13,057,473 during Period 1) and 26.3% (3,507,120 of 13,337,407 during Period 2) of all canine patients receiving HWP. Of the included practices, 324 (7.0%) fit the definition of Group 1 (New PH 12 Users), 1,406 (30.4%) fit the definition of Group 2 (PH 6 to PH 12 Users), 627 (13.5%) fit the definition of Group 3 (PH 6 Only Users), and 2,258 (48.9%) fit the definition of Group 4 (Non-PH Users). Patients within these practices with mixed product purchases (meaning patients receiving both PH and a MHWP within the same Period) were excluded for clarity purposes. Patients purchasing a mix of MHWP products (purchasing Heartgard Plus and Interceptor, for example) within the same period were included.

An average of only 25% of all canine patients seen at these clinics during the examination period received some sort of heartworm protection in this study. In the total study population, the growth in canine heartworm patients (6.3%) was greater than the overall canine patient growth (2.1%) year over year (YOY; Period 1 vs. Period 2) (Table 1).

**Table 1:** Comparison of overall practice revenue, period 1 vs period 2.

*Measure*	*Period 1*	*Period 2*	*Percent change*
*Overall Clinic Revenue*	$7,566,477,148	$8,107,325,934	7.1%
*Canine Clinic Revenue*	$5,954,974,077	$6,401,349,700	7.5%
*Canine HWP Revenue*	$229,024,080	$257,157,037	12.3%
*Canine Clinic Patients*	13,057,473	13,337,407	2.1%
*Canine HWP Patients*	3,299,200	3,507,120	6.3%

All Groups–Key Metrics: Total for All Groups. Number of Practices: 4,615 (100% of sample)

Of all the canine patients that purchased heartworm preventives from the veterinary practice, the proportion that were fully purchase compliant (i.e., purchased 12 months coverage) was 36.5% in Period 1 and 40.1% in Period 2, representing a 9.8% growth in fully purchase compliant patients YOY. However, the percent of preventive patients purchasing monthly heartworm preventives (MHWP) that were fully compliant dropped from 35.9% in Period 1 to 33.9% in Period 2; and the percent of preventive patients purchasing PH 6 that were fully compliant (i.e., purchased two doses in the same Period) also dropped from 39.9% to 35.5%. PH 12’s 12-month purchase compliance was inherently 100% (since the product provides 12 months of continuous protection with one dose) (Table 2) and was responsible for all of the observed growth in canine preventive patients protected for a full 12 months year over year across all study groups.

**Table 2:** 

Product	1–3 months	4–5 months	6 months	7–9 months	10–11 months	12+ months	Total	12+ months compliant	95% CI
MHWP	594,559	106,390	936,691	178,065	55,125	1,048,002	2,918,832	35.9%	35.69–36.12%
PH6			284,199			188,477	472,676	39.9%	39.69–0.05%
PERIOD 1 TOTAL	594,559	106,390	1,220,890	178,065	55,125	1,236,479	3,391,508	36.5%	36.25–36.66%
MHWP	662,252	85,879	924,566	187,642	58,522	982,441	2,901,302	33.9%	33.65–34.07%
PH12						324,001	324,001	100.0%	99.66–100.34%
PH6			202,864			111,823	314,687	35.5%	35.33–35.74%
PERIOD 2 TOTAL	662,252	85,879	1,127,430	187,642	58,522	1,418,265	3,539,990	40.1%	39.86–40.27%
PERCENT CHANGE	11.4%	-19.3%	-7.7%	5.4%	6.2%	14.7%	4.4%	9.9%	

All Groups: 12+ months compliance across HWP patients (note: patients with mixed product transactions have been excluded for clarity)

The percent of canine preventive patients protected for *more than* 6 months (i.e., 7–12+ months) was 43.3% in Period 1 and 47.0% in Period 2, a 3.7 percentage point increase, representing an 8.5% growth YOY. In Period 1 and Period 2 the percent of patients purchasing monthly preventives that purchased *more than* 6 months of protection represented only 43.8% and 42.3% in Period 1 and Period 2, respectively. Therefore, less than half of canine patients purchasing monthly heartworm preventives receive more than 6 months of protection, and no growth in this subset of patients was observed YOY. PH 12 was responsible for all the observed growth in canine patients protected for more than 6 months. See Table 2.

#### Group 1: New PH 12 users

In the 324 practices that constituted Group 1, New PH 12 Users (practices that carried only MHWP and no PH in Period 1, but brought on PH 12 in Period 2), the percent of canine patients purchasing heartworm preventives grew more than overall canine clinic patient growth (9.2% vs 2.6%), showing a higher percent of patients being protected YOY (Table 3). The proportion of transactions sourced from PH 12 increased from 0 to 17%.

**Table 3:** Comparison of practice revenue, period 1 vs period 2, New PH 12 Users.

MEASURE	PERIOD 1	PERIOD 2	PERCENT CHANGE
**OVERALL CLINIC REVENUE**	$607,589,766	$653,125,112	7.5%
**CANINE CLINIC REVENUE**	$476,087,211	$512,751,047	7.7%
**CANINE HWP REVENUE**	$17,915,860	$20,629,591	15.1%
**CANINE CLINIC PATIENTS**	1,012,096	1,038,094	2.6%
**CANINE HWP PATIENTS**	255,959	279,559	9.2%

Group 1: New PH 12 Users.

Within Group 1, the percent of canine preventive patients that purchased a full 12 months of preventive increased from 40.0% in Period 1 to 46.0% in Period 2, a six-percentage point increase and a 15% growth YOY, despite the observation that full compliance in monthly heartworm preventive patients declined. Thus, the 15% growth in fully compliant patients in these clinics was driven by PH 12 (Table 4). The percent of canine preventive patients that purchased more than 6 months protection increased from 48.0% to 53.0%. This growth was entirely due to the addition of PH 12 to the practice as the percent of monthly users purchasing more than 6 months of preventive declined slightly.

**Table 4:** Patient level comparison of doses purchased Period 1 vs Period 2. New PH 12 Users.

PRODUCT	1–3 months	4–5 months	6 months	7–9 months	10–11 months	12+ months	Total	12+ months compliant	95% CI
MHWP	45,519	7,587	78,394	15,173	5,058	101,154	252,885	40.0%	39-75-40.25%
PERIOD 1 TOTAL	45,519	7,587	78,394	15,173	5,058	101,154	252,885	40.0%	39-75-40.25%
MHWP	46,999	8,294	71,881	13,823	5,529	88,469	234,995	37.6%	37.40–37.90%
PH12						33,176	33,176	100.0%	98.93–101.08%
PH6			2,765				2,765	0%	NA
PERIOD 2 TOTAL	46,999	8,294	74,646	13,823	5,529	127,174	276,465	46.0%	45.75–46.25%
PERCENT CHANGE	3.30%	9.30%	-4.80%	-8.90%	9.30%	25.70%	9.30%	15.00%	

Group 1: New PH 12 Users.

#### Group 2: PH 6 to PH 12 users

In the 1,406 practices that constituted Group 2, PH 6 to PH 12 Users (practices that carried MHWP and PH 6 in Period 1, and brought on PH 12 in Period 2), the number of canine HWP patients grew more than overall canine clinic patient growth (7.2% vs 2.0%), showing a higher percent of patients being protected YOY (Table 5). The proportion of transactions sourced from PH increased by 7%, revealing that more patients received doses of injectable moxidectin once the annual form was added to the formulary.

**Table 5:** All practice revenue comparison Period 1 vs Period 2. PH 6 to PH 12 Users.

MEASURE	PERIOD 1	PERIOD 2	PERCENT CHANGE
**OVERALL CLINIC REVENUE**	$2,625,772,822	$2,863,940,804	9.1%
**CANINE CLINIC REVENUE**	$2,128,120,347	$2,326,099,656	9.3%
**CANINE HWP REVENUE**	$94,399,160	$113,005,144	19.7%
**CANINE CLINIC PATIENTS**	4,722,132	4,817,261	2.0%
**CANINE HWP PATIENTS**	1,343,794	1,439,926	7.2%

Group 2: PH 6 to PH 12 Users.

Within Group 2, the percent of canine preventive patients that purchased a full 12 months of preventive increased from 37.8% to 48.0% YOY, a 10-percentage point increase and a 26.9% growth YOY. MHWP patients and PH 6 patients in this group that purchased a full 12 months declined YOY. The incremental growth of canine patients purchasing 12 months was entirely driven by PH 12 (Table 6), as was the incremental growth in percent of canine preventive patients that purchased more than 6 months protection (43.6% to 53.6%). This group experienced the highest compliance and revenue growth of any group in this study.

**Table 6:** Patient level comparison of doses purchased Period 1 vs Period 2. PH 6 to PH 12 Users.

Product	1–3 months	4–5 months	6 months	7–9 months	10–11 months	12+ months	Total	12+ months compliance	95% CI
MHWP	200,233	34,803	274,683	56,539	18,369	342,532	927,159	36.9%	36.82–37.07%
PH6			220,600			147,365	367,965	40.0%	39.84–40.25%
PERIOD 1 TOTAL	200,233	34,803	495,283	56,539	18,369	489,897	1,295,124	37.8%	37.72–37.93%
MHWP	211,455	36,180	252,494	56,517	18,447	295,528	870,621	33.9%	33.82–34.07%
PH12						290,825	290,825	100.0%	99.64–100.36%
PH6			126,885			61,814	188,699	32.8%	32.50–33.02%
PERIOD 2 TOTAL	211,455	36,180	379,379	56,517	18,447	648,167	1,350,145	48.0%	47.89–48.12%
PERCENT CHANGE	5.60%	4.00%	-23.40%	0.00%	0.40%	32.30%	4.20%	26.9%	

Group 2: PH 6 to PH 12 Users.

#### Group 3: PH 6 only users

In the 627 practices that constituted Group 3, PH 6 Only Users (practices that carried MHWP and PH 6 in Period 1 and Period 2), the number of canine HWP patients grew more than overall canine clinic patient growth (8.9% vs. 2.7%), showing a higher proportion of patients being protected YOY (Table 7). The proportion of transactions sourced from PH 6 increased by 5%.

**Table 7:** All practice revenue comparison Period 1 vs Period 2. PH 6 only users.

MEASURE	PERIOD 1	PERIOD 2	PERCENT CHANGE
**OVERALL CLINIC REVENUE**	$944,703,634	$1,013,808,137	7.3%
**CANINE CLINIC REVENUE**	$750,915,296	$807,144,973	7.5%
**CANINE HWP REVENUE**	$29,525,105	$32,916,603	11.5%
**CANINE CLINIC PATIENTS**	1,711,200	1,757,209	2.7%
**CANINE HWP PATIENTS**	425,288	463,199	8.9%

Group 3: PH 6 Only Users.

Within Group 3, the percent of canine preventive patients that purchased a full 12 months of preventive declined from 35.8% to 34.6%, a one percentage point drop, and a 3.5% decline YOY. PH 6 patients that purchased a full 12 months increased slightly from 39.3% to 40.6%, however, MHWP patients in this group that purchased a full 12 months dropped from 34.7% to 32.3%, leading to an overall decline in compliance in this group YOY (Table 8).

**Table 8:** Patient level comparison of doses purchased Period 1 vs Period 2. PH 6 only users.

Product mixes	1–3 months	4–5 months	6 months	7–9 months	10–11 months	12 months	Total	12+ months compliance	95% CI
MHWP	67,861	11,293	98,028	19,245	5,871	107,411	309,709	34.7%	34.47–34.89%
PH6			63,599			41,112	104,711	39.3%	38.88–39.64%
PERIOD 1 TOTAL	67,861	11,293	161,627	19,245	5,871	148,523	414,420	35.8%	35.66–36.02%
MHWP	79,613	12,676	99,505	21,315	6,457	104,850	324,416	32.3%	32.12–32.52%
PH6			73,214			50,009	123,223	40.6%	40.23–40.94%
PERIOD 2 TOTAL	79,613	12,676	172,719	21,315	6,457	154,859	447,639	34.6%	34.42–34.77%
PERCENT CHANGE	17.30%	12.20%	6.90%	10.80%	10.00%	4.30%	8.00%	-3.5%	

Group 3: PH 6 Only Users.

#### Group 4: Non-PH users

In the 2,258 practices that constituted Group 4, Non-PH Users (practices that carried MHWP only in Period 1 and Period 2), the number of canine HWP patients grew only slightly compared to overall canine patient growth (3.9% vs. 2.0%), showing only a slightly higher percent of patients being protected YOY (Table 9).

**Table 9:** All practice revenue comparison Period 1 vs Period 2. Non-PH users.

MEASURE	PERIOD 1	PERIOD 2	PERCENT CHANGE
**OVERALL CLINIC REVENUE**	$3,388,410,926	$3,576,451,881	5.5%
**CANINE CLINIC REVENUE**	$2,599,851,224	$2,755,354,024	5.9%
**CANINE HWP REVENUE**	$87,183,956	$90,605,698	3.9%
**CANINE CLINIC PATIENTS**	5,612,045	5,724,843	2.0%
**CANINE HWP PATIENTS**	1,274,159	1,324,436	3.9%

Group 4: Non-PH Users.

Within Group 4, the percent of canine preventive patients (all MHWP patients) that purchased a full 12 months of preventive declined from 34.8% to 33.5%, a one percentage point drop, and a 3.7% decline YOY–despite being the only heartworm preventive option in these practices (Table 10). Likewise, the proportion of canine preventive patients that purchased more than 6 months protection decreased slightly from 42.7% to 42.0%.

**Table 10:** Patient level comparison of doses purchased Period 1 vs Period 2. Non-PH users.

Product	1–3 months	4–5 months	6 months	7–9 months	10–11 months	12+ months	Total	12+ months compliance	95% CI
MHWP	280,946	52,707	485,586	87,108	25,827	496,905	1,429,079	34.8%	34.67–34.87%
PERIOD 1 TOTAL	280,946	52,707	485,586	87,108	25,827	496,905	1,429,079	34.8%	34.67–34.87%
MHWP	324,185	28,729	500,686	95,987	28,089	493,594	1,471,270	33.5%	33.46–33.64%
PERIOD 2 TOTAL	324,185	28,729	500,686	95,987	28,089	493,594	1,471,270	33.5%	33.46–33.64%
PERCENT CHANGE	15.40%	-45.50%	3.10%	10.20%	8.80%	-0.70%	3.00%	-3.70%	

Group 4: Non-PH Users.

#### Revenue

Across the entire study population, heartworm preventive revenue increased by 12.3% YOY, and the average canine patient transaction including heartworm preventive from $69.42 per canine patient in Period 1 to $73.32 in Period 2.

In Group 1 (New PH 12 Users) canine HWP revenue increased from $17,915,860 to $20,629,591 (15.1% increase, outpacing the overall clinic revenue increase in this group of 7.5%) (Table 3). HWP revenue increased from $70.00 per canine patient in Period 1 to $73.79 in Period 2. In Group 2 (PH 6 to PH 12 Users) canine HWP revenue increased by 19.7% (outpacing the overall clinic revenue increase in this group of 9.1%) (Table 5), and HWP revenue increased from $70.25 per canine patient to $78.48. In Group 3 (PH 6 Only Users) canine HWP revenue increased by 11.5% (outpacing total clinic revenue increase in this group of 7.3%) (Table 7), and HWP revenue increased from $69.42 per canine patient to $71.06. In Group 4, (Non-PH Users) canine HWP revenue increased by 3.9% (underperforming total clinic revenue increase in this group of 5.5%) (Table 8). HWP revenue remained the same at $68.41 per dog in both Periods.

A summary of YOY compliance and revenue growth or decline observed for each Group is provided in Table 11.

**Table 11:** Summary of year over year growth or decline for all groups.

*Measure*	*ALL*	*Group 1 New PH Users*	*Group 2 PH 6/12 Users*	*Group 3 PH 6 Only*	*Group 4 Non-PH Users*
*Overall clinic revenue*	**7.1%**	7.5%	9.1%	7.3%	5.5%
*Canine clinic revenue*	**7.5%**	7.7%	9.3%	7.5%	5.9%
*Canine HWP revenue*	**12.3%**	15.1%	19.7%	11.5%	3.9%
*Canine clinic patients*	**2.1%**	2.6%	2.0%	2.7%	2.0%
*Canine HWP patients*	**6.3%**	9.2%	7.2%	8.9%	3.9%
*12-month compliance*	**9.9%**	15.0%	26.9%	-3.5%	-3.7%
*>6 month compliance*	**8.5%**	10.4%	22.8%	-2.6%	-1.6%

## Discussion

### Compliance

An average of only 25% of canine patients seen at these clinics during the 2 year observation period received some sort of heartworm protection in this study, consistent with findings of previous studies [1]. This lack of compliance is deemed the main reason for worsening canine heartworm spread [1, 3, 5]. On a positive note, the percent of canine patients receiving heartworm protection in this study increased 6.3% YOY across this study population of over 13 million medicalized dogs, outpacing overall canine patient growth (2.1%). Practices that brought on PH 12 experienced over 6% growth in the proportion of patients receiving heartworm preventive, whereas practices that continued to carry only monthly preventives grew the proportion of patients receiving heartworm preventive at a lower rate of 2.2% YOY.

Injectable heartworm prevention puts compliance in the veterinarian’s control. Thus, PH 12 patients were expected to drive an increase in months protected. The observed growth in percent of patients receiving heartworm protection in practices with PH 12 vs. non-PH clinics was interesting, as one might assume owners will be less likely to purchase a single injection vs. one or more monthly preventives. Although the cost of PH 12 is similar to annual doses of monthly heartworm preventive, owners purchasing the latter have the option of buying single doses at a time. But this bias did not appear to impact sales. Practices only offering MHWP had a lower proportion of their patients on any amount of heartworm preventive than PH practices, and PH 12 drove growth in percent of patients on prevention in addition to months protected in those patients.

Of canine patients purchasing heartworm preventives, compliance is historically poor [1, 5]. Only one-third (34.8%) of dogs purchasing monthly heartworm prevention in this study (roughly 1 in 10 canine patients) purchased 12 months over the course of the year (i.e., were fully compliant), in single or multiple transactions. These findings are consistent with previous studies [1]. Further, only 42.9% of MHWP patients were protected for more than 6 months of the year, revealing that the average monthly preventive user purchases 6 doses or less. Given that many monthly heartworm preventives on the market today are labeled to prevent heartworm disease if given for several months past the dog’s last mosquito exposure [6], 6 months of heartworm prevention even in temperate climates of the US is not adequate for protection [3].

PH 12 provides 12 months of continuous heartworm protection for all patients receiving it, and thus renders its patients fully compliant according to AHS recommendations. This study presents preliminary findings. It remains to be determined what the compliance for this product will be in the US over multiple years. Compliance amongst monthly heartworm preventive patients did not increase in this study year over year. A similar study based on Australian data found that Single-year compliance with ProHeart SR-12 was 92.8–96.9% vs. 26.9–36.5% for dogs receiving MHWP products and multiple year compliance was 76.7% for ProHeart SR-12 and 24.4% for MHWP medications [9]. Australia has a lower age of first use for injectable moxidectin (12 weeks), compared to the US, where PH 12 is approved for use in dogs 1 year of age and older.

In both Group 1 and 2 (New PH 12 Users and PH 6 to PH 12 Users, respectively), implementation of PH 12 improved overall compliance dramatically (double digit growth). Practices that were new to PH (Group 1) saw substantial growth in the proportion of canine preventive patients that were fully compliant for 12 months (15%). In practices with MHWP and PH 6 that brought on PH 12 (Group 2), PH 12 drove growth in patients protected for 12 months (26.9%).

Patient compliance declined in both groups that did not bring on PH 12. Non-PH Users (Group 4) experienced a decline of 3.7% in fully compliant patients. In Group 3 (PH 6 Only Users), PH 6 was responsible for higher 12-month compliance rates compared to monthly users, but overall growth in 12-month compliance rates in this group was not seen due to declines in monthly users. This stagnation of monthly preventive compliance rates has been documented previously [1].

In a previous retrospective study, the average proportion of dogs that were fully compliant (2 x 6-month injections) of PH 6 administered 5–7 months apart was 51.7%, whereas, the average number of dogs compliant with purchasing 6 doses of MHWP preventives < 7 months apart was 32.8% [10]. The 12-month compliance rate of PH 6 users in this study was not as high (35.5–39.9%), possibly attributable to the inclusion of a 12-month product on the market as well as the fact that the previous study examined repeat visits up to 7 months for the second injection. Additionally, in that study, dogs receiving PH 6 had a higher proportion of patients with repeat injections within 12 months between 2014–2017, with 68% retention rate after 4 years. In comparison, the 6 dose MHWP cohort retention rate dropped to 55% after 4 years. That study concluded that dogs receiving PH 6 had better compliance and superior practice retention compared with MHWP products [10].

PH 6 is labeled for use in dogs 6 month of age or older and will likely be implemented as a bridge preventive until the patient is old enough (12 months of age) to receive PH 12. Therefore, PH 6 12-month compliance rates in practices bringing on PH 12 were not surprising. However, once PH 12 was brought on board, it resulted in higher compliance growth for these practices than PH 6 practices that did not bring on PH 12. In fact, PH 6 practices that brought on PH 12 experienced the highest compliance and revenue growth of any group in this study. This is possibly due to the fact these practices were already well versed in implementing injectable heartworm preventive protocols, and therefore were able to maximize the benefits of PH 12 more quickly than practices that were new to PH.

Despite the smaller proportion of practices in this study carrying PH, nationwide in its first year on the market PH 12 incrementally grew heartworm preventive compliance (months protected) of the total study canine patient population. Specifically, PH 12 was responsible for the growth in fully compliant patients (a YOY growth of 9.9% across the total study population), as well as growth in patients protected greater than 6 months (Table 2). Given that nearly half of all clinics in this study were in Group 4 (Non-PH Users), the growth in canine heartworm preventive compliance rates were achieved due to PH 12 despite MHWP compliance losses in Group 4, as well as MHWP compliance declines or stagnation observed in all groups.

### Revenue

Across all groups, canine heartworm preventive revenue increased by 12.3% YOY. In Group 1, New PH 12 Users, heartworm preventive revenue increased by 15.1% (outpacing the total clinic revenue increase in this group of 7.5%) in comparison to non-PH practices (Group 4) wherein heartworm preventive revenue increased by only 3.9% (underperforming clinic revenue increase in this group of 5.5%). In Group 2, practices with PH 6 and MHWP that brought on PH 12, canine heartworm preventive revenue increased by 19.7%, compared to practices with PH 6 that did not add PH 12 to their practice (Group 3), wherein preventive revenue increased by 11.5%. Not only was this revenue growth in practices with PH 6 and 12 due to increased compliance and patients YOY, but pharmacy revenue from PH administration remains in the veterinary practice as PH cannot be sourced by the owner through online retailers. In contrast, a prescription of monthly heartworm prevention to a non-compliant owner is more likely to result in a purchase of less than 12 monthly doses. Thus, it makes sense that practices implement PH 12 into their pharmacy to promote compliance where it is lacking, experienced higher preventive, and overall clinic revenue increases YOY.

## Conclusion

While there are different reasons veterinarians chose to implement various heartworm preventive products, all with their own benefits and drawbacks, when considering the importance of patient protection against potentially deadly heartworm disease, unless the prevention is in the patient throughout the epidemiologic risk timeframe, it will be less effective. The average monthly heartworm preventive purchaser in this study purchased six months or less of preventive, and this compliance rate declined YOY. Preventive compliance is essential in the prevention of this disease. If failure to fully protect patients against this disease continues, we will likely continue to see a rise in cases and geographical spread.

This data shows that veterinary practices implementing long-acting injectable heartworm preventive were more successful in increasing the proportion of their patients protected, achieved higher monthly and full year compliance in their patients, and received a higher increase in heartworm preventive revenue YOY, compared to practices that did not include PH 12 in their pharmacy. This preliminary data exemplified PH 12’s impact on the canine heartworm preventive market and canine heartworm disease prevention. PH 12 practices developed a 23% higher full compliance rate than did practices without PH 12. Lastly, despite the smaller proportion of practices in this study carrying PH 12, in its first year on the market PH 12 single-handedly grew the full compliance rate of the total canine patient population on heartworm prevention by nearly 10%. Long-acting injectable heartworm prevention is therefore a powerful mitigation tool in curbing the spread of this deadly infectious disease by helping to fill existing preventive compliance gaps.
